# Up to the chest in bowels: case of strangulated right diaphragmatic hernia with paraesophageal hernia in a non-trauma patient

**DOI:** 10.1093/jscr/rjae162

**Published:** 2024-04-03

**Authors:** Elizabeth R Maginot, Jason Lizalek, Mike Matos

**Affiliations:** Department of Surgery, College of Medicine, University of Nebraska Medical Center, 42nd and Emile Streets, Omaha, NE 68198, United States; Department of Surgery, College of Medicine, University of Nebraska Medical Center, 42nd and Emile Streets, Omaha, NE 68198, United States; Department of Surgery, Division of Acute Care Surgery, University of Nebraska Medical Center, Omaha, NE 68198, United States

**Keywords:** Bochdalek hernia, adult, hiatal hernia, cardiopulmonary comorbidities

## Abstract

A Bochdalek hernia is a rare congenital diaphragmatic hernia often diagnosed in infancy and classically occurring on the left side. We report a case of a 78-year-old female who presented with a right-sided posterolateral diaphragmatic hernia containing multiple loops of bowel with evidence of ischemia as well as a type 4 paraesophageal hernia. The stomach was rotated on the organoaxial plane, and the duodenum was within the mediastinum. The patient was taken emergently for an exploratory laparotomy. A posterolateral hernia defect containing 50 cm of strangulated small bowel was identified and resected, a primary stapled enteroenterostomy was performed and the hernia defect was repaired primarily. The stomach was reduced, a primary crura repair was performed, and gastropexy was performed with a gastrojejunostomy tube. The patient was transferred to the intensive care unit, and subsequently extubated, enteral feeds were initiated, and had anticipated discharge to a skilled nursing facility. This case highlights an uncommon atraumatic presentation of an adult with a congenital diaphragmatic hernia. Its rarity is further denoted due to its right-sided laterality and strangulated small bowel as the usual herniated abdominal organs are the liver or colon.

## Introduction

A Bochdalek hernia is a congenital diaphragmatic hernia that is located in the posterolateral lumbocostal triangle. Congenital diaphragmatic hernias are a rare pathology, occurring in ~1 out of 2500 births [1]. The incidence of adult Bochdalek hernias is only estimated to be ~0.17% [2].

We present an acute case of a nontraumatic right-sided Bochdalek diaphragmatic hernia in an elderly patient with a concomitant type 4 hiatal hernia. Although there are similarities between this case presentation and other presentations of adult Bochdalek hernias [3], there are multiple factors making this case presentation rare. Typically arising on the left side, Bochdalek hernias usually contain fat or omentum and do not commonly lead to symptoms in late life [2]. The acuity of presentation, corresponding hiatal hernia, and laterality create a rare and interesting presentation.

## Case report

We present the case of a 78-year-old female patient hospitalized for progressive dyspnea and sudden-onset epigastric abdominal pain. She was in mild distress upon presentation. Vital signs showed tachycardia into the 140–150 s with an irregularly irregular rhythm, temperature 36.7 C, respiratory rate 15 breaths per minute, and blood pressure 104/57 mm Hg.

On physical examination, her abdomen was tender to palpation in left upper quadrant and epigastric region. Exam without signs of peritonitis. Her laboratory work-up showed leukocytosis (white blood cell count 14 100/uL), anemia with hemoglobin of 9.9 g/dL, and mildly elevated lactic acid at 1.9 mmol/L.

Her medical history was notable for atrial fibrillation. She also had findings of a type 4 hiatal hernia and chronic obstructive pulmonary disease and interstitial lung disease for which she was on 2–3 L of oxygen at baseline at home.

**Figure 1:**
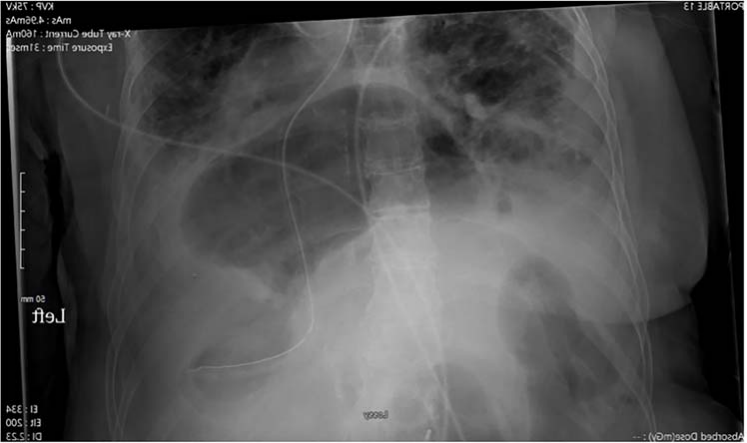
X-ray of the abdomen: anterior–posterior portable X-ray of the abdomen showing bowel gas pattern in the right upper abdomen and a large hiatal hernia with gaseous distention of the stomach.

**Figure 2:**
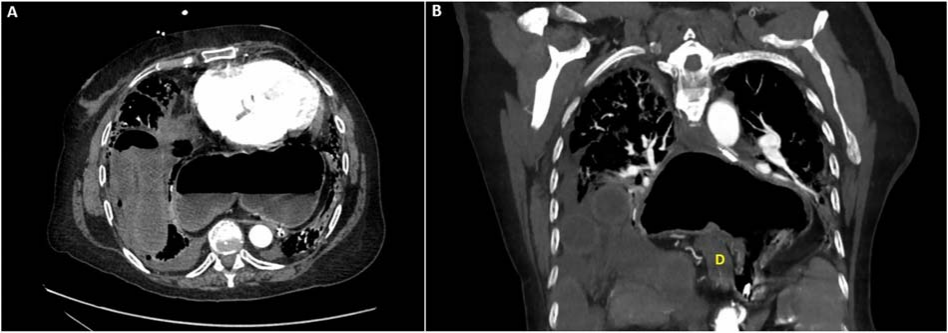
Computed tomography of the chest and abdomen: (A) axial section demonstrating incarcerated small bowel within the right hemithorax as well as stomach within the posterior mediastinum. (B) Coronal section demonstrating incarcerated small bowel within the right hemithorax above the liver as well as hiatal hernia containing stomach in an organoaxial rotation as well as the duodenum (D).

Abdominal radiography demonstrated a large hiatal hernia with intrathoracic stomach and concerns for gastric volvulus and partially imaged bowel gas within the right upper abdomen (Fig. 1). Computerized tomography of the chest and abdomen showed a large distended hiatal hernia with organoaxial rotation of the stomach, gastric distention with fluid and gas raising concern for partial obstruction. The right hemithorax contained multiple mildly dilated fluid-filled loops of small bowel with a prominent infiltrate of mesentery and decompressed bowel distally concerning for strangulation by a posterior lateral diaphragmatic hernia. There was no evidence of pneumatosis (Fig. 2).

The patient was taken emergently to the operating room for exploratory laparotomy. The operation revealed 50 cm of frankly necrotic bowel that was reduced from a posteriolateral 4 cm diaphragmatic hernia. This was resected and a side-to-side stapled anastomosis was performed. The diaphragmatic hernia was closed primarily in a transverse fashion with interrupted horizontal mattress #1 braided nonabsorbable sutures and a 32 French thoracostomy chest tube was placed.

The stomach was then easily reduced into the abdominal cavity and the large crural defect was primarily closed using #1 braided nonabsorbable sutures in a figure-of-eight pattern. This was not closed tightly due to the diminutive nature of the muscle and plans for future gastropexy. A Stamm gastrojejunostomy tube was performed as a gastropexy as well as provide a route for postoperative enteral nutrition.

The patient was admitted to the intensive care unit postoperatively and extubated. She had a prolonged hospitalization given her underlying pulmonary disease and recovery from an open, emergent operation. She was discharged to a skilled nursing facility.

## Discussion

We report a rare case of a right-sided Bochdalek diaphragmatic hernia with a concomitant type 4 hiatal hernia in a 78-year-old woman without prior trauma. Cross-sectional imaging revealed a large distended hiatal hernia with organoaxial rotation of the stomach and right posterior lateral diaphragmatic hernia with strangulated small bowel. Urgent exploratory laparotomy revealed necrotic bowel contributing to her acute clinical picture. The type 4 hiatal hernia and gastric volvulus emphasize the uniqueness of this case presentation.

The foramen of Bochdalek is a 2 cm × 3 cm opening in the posterior aspect of the diaphragm, which typically closes around week 8 of gestation [4]. A hernia forms when the communication between the pleuroperitoneal canal and peritoneal cavity does not close [5]. Because the right canal closes before the left, left-sided Bochdalek hernias are far more common (85%) when compared to right-sided hernias [4].

This case highlights the interesting implications of intrathoracic hernias and corresponding cardiopulmonary comorbidities. Our patient presented in atrial fibrillation with rapid ventricular response. In 2016, Roman *et al.* showed an increased odds ratio of arrhythmia in patients with hiatal hernias [6]. It also illustrated that larger hernias have greater odds of having a cardiac arrhythmia compared to smaller ones [6]. In 2020, Akita *et al.* reviewed 55 operative case reports of Bochdalek hernias and found that cardiopulmonary comorbidities were more common in cases with bowel strangulation [7]. Intraoperatively, the patient’s atrial fibrillation was rate controlled following the reduction of her Bochdalek hernia. The patient required prolonged intubation following operative management and was extubated on postoperative Day 3.

In addition, the acute late-onset nature of this hernia is very unusual for Bochdalek hernia; although, other case reports have been noted [4, 8]. Upon literature review, we were unable to find a presentation with concomitant hiatal hernia or gastric volvulus adding to the uniqueness of this case.

Adult presentation of symptomatic Bochdalek hernia is a rare presentation. The right-sided nature and large hiatal hernia with gastric volvulus adds to the complexity and rarity of this case presentation. It highlights the implications of large Bochdalek hernia in an adult patient and the necessity of repair, if diagnosed.
